# Bridging Experiment and AI: SAXS-Driven Molecular Dynamics Refinement of Predicted Biomolecular Structures

**DOI:** 10.1063/4.0000956

**Published:** 2025-10-27

**Authors:** Kriti Chopra, Lewis A Rolband, James Byrnes, Kirill A Afonin, Hubertus JJ van Dam

**Affiliations:** 1 Brookhaven National Laboratory, Upton, New York, USA; 2 University of North Carolina Charlotte, Charlotte, North Carolina, USA; 3 Universität Duisburg-Essen, Essen, Essen, Germany

## Abstract

SAXS/WAXS data collection provides valuable insights into biomolecular structure and dynamics in solution, yet transforming this experimental data into accurate three-dimensional models remains challenging. We have developed a comprehensive workflow that integrates structure prediction methods, including AI prediction models such as AlphaFold, with SAXS-driven molecular dynamics (SAXS-MD) to characterize complex biomolecular assemblies. Using RNA nanoparticles (NANPs) and complex biomolecular assemblies as examples, we demonstrate how our computational pipeline reveals conformational dynamics that remain hidden in static structural techniques. The workflow combines experimental SAXS constraints with predicted structure models and refines them through SAXS-MD simulations, leveraging high-performance computing resources to achieve the necessary simulation timescales and sampling efficiency. This integrated approach identifies key dynamic regions, characterizes conformational ensembles, and elucidates structure-function relationships in systems with inherent flexibility. The synergy between experimental data, AI-driven predictions, and HPC-enabled simulations enhances our structural biology toolkit, offering complementary insights that bridge the gap between static models and dynamic biological processes.

